# Anxiety and depression in patients with breast cancer undergoing radiotherapy: the role of intelligence, life history, and social support—preliminary results from a monocentric analysis

**DOI:** 10.1007/s00066-022-01904-7

**Published:** 2022-03-03

**Authors:** Marc D. Piroth, Silvia Draia, Jehad Abu Jawad, Martina Piefke

**Affiliations:** 1 Department of Radiation Oncology, Helios University Hospital Wuppertal, Faculty of Health, Witten/Herdecke University, Heusnerstr. 40, 42283 Wuppertal, Germany; 2 Neurobiology and Genetics of Behavior, Department of Psychology and Psychotherapy, Faculty of Health, Witten/Herdecke University, Alfred-Herrhausen-Str. 50, 58448 Witten, Germany

**Keywords:** Breast cancer radiotherapy, Anxiety, Depression, Intelligence, Social support

## Abstract

**Purpose:**

It is known that the diagnosis of breast cancer often causes anxiety and depression. Radiotherapy of the breast as an obligatory part of a breast-conserving treatment concept can markedly increase these psychological symptoms in many, but not all patients. In this clinical observational study, we aimed at identifying cognitive, health-related and social factors that may either enhance or reduce the emergence of anxiety and depression.

**Methods:**

Using a longitudinal study design with 25 women (mean age: 52.9 years; SD = 10.6; age range 29–70 years) with a first diagnosis of nonmetastatic breast cancer, measures of anxiety, depression, situational emotional states, intelligence, and aspects of social frameworks were assessed before, during, and after radiotherapy of the breast. At 4 time-points, standard and self-constructed questionnaires were used to assess the course of anxiety and depressive symptoms across the radiotherapy intervention.

**Results:**

We found that anxiety is highest immediately before the start of radiation therapy, while the anxiety level was lowest on the day that therapy was completed. Anxiety and depression were enhanced in women with a lifetime history of chronic diseases at all time points of measurement. Moreover, women with high intelligence and low social support had stronger symptoms of depression than women with low intelligence and a stable family background at some time points of measurement. The degree of anxiety was neither related to intelligence nor to social support.

**Conclusion:**

For the first time, we demonstrate empirical pilot data on cognitive and social modulators of anxiety and depression in women with breast cancer over the course of radiotherapy. Our results may help to optimize clinical procedures and thereby reduce symptoms of anxiety and depression in these patients.

**Supplementary Information:**

The online version of this article (10.1007/s00066-022-01904-7) contains supplementary material, which is available to authorized users.

## Introduction

Breast cancer is the most common type of cancer and a leading cause of cancer death in women [1]. The median age at diagnosis is 62 years but 18% of the women are ≤ 49 years old [2]. Every eighth woman will develop breast cancer in the course of her life; thus the treatment of breast cancer is a burden for numerous women.

It is known that the diagnosis of breast cancer often causes anxiety and depression. Fear of future becomes a main problem after diagnosis and during the treatment of breast cancer [3, 4]. Tsaras et al. reported prevalences of depression and anxiety of 38.2% and 32.2% in breast cancer patients [5]. In a study performed by Civilotti et al., even 52.1% of patients affected by breast cancer showed anxiety symptoms [4]. The levels of anxiety and depression frequently increase due to the expectation of progression of the disease, pain, and suffering from cancer treatment [4]. It has been shown that depression or other emotional distress like stressful life experiences may exert negative effects on cancer mortality and also cancer survival [6].

Breast cancer treatment is mostly based on three pillars: surgery, radiotherapy and systemic treatment like chemotherapy, immunotherapy and/or hormonal treatment. Radiotherapy of the breast is an obligatory part of a breast-conserving treatment concept and can lead to a significant improvement in locoregional control, breast cancer-specific survival, and, in long-term follow-up, also in overall survival [7]. Despite the always better and increasingly gentle techniques [8], breast cancer radiotherapy influences aspects of quality of life such as physical well-being [9, 10]. Furthermore, despite the very positive influence on the prognosis or cure, radiotherapy of the breast may lead to a substantial increase of anxiety and depression. Stiegelis et al. showed that 21–54% of patients develop feelings of anxiety and 12–31% develop symptoms of depression during the course of radiotherapy [11]. Lewis et al. found that anxiety, recorded using visual analogue scales (VAS), may be highest at the beginning of the radiotherapy, especially in the radiotherapy simulation phase and the first radiotherapy sessions. Afterwards, it drops off significantly [12]. Based on these findings, Lewis et al. proposed that the availability of appropriate information for the patient before starting radiotherapy can reduce anxiety [13].

Furthermore, it has been shown that demographic and health-related factors may modulate the emergence and severity of anxiety and depression after a diagnosis of breast cancer. For example, Tsaras et al. conducted a descriptive study including patients who were treated with different oncological therapies before and who had thereafter regular follow-up’s. [5]. The authors showed that the place of residence, religion and current activity-symptoms burden were significant predictors of anxiety and also depression [5]. To date, it is unknown whether other demographic, health-related, and personal as well as further cognitive factors exist that also contribute to the development of symptoms of anxiety and/or depression during radiotherapy.

In the present clinical observational study we aimed at investigating cognitive and demographic factors that may either enhance or reduce the emergence of anxiety and depression before, during, an immediately after radiation therapy. In particular, we were interested in the role of the patients’ level of intelligence, facets of their life history, and their support from family members. We expected that patients with higher levels of intelligence would show lower anxiety and depression in the course of radiotherapy than patients with low levels of intelligence. Moreover, we hypothesized that several forms of social support would generally reduce symptoms of anxiety and depression in the patients.

## Methods

### Participants

In all, 25 women who underwent adjuvant breast cancer radiotherapy between October 2018 and March 2019 in the Department of Radiation Oncology, Helios University Hospital Wuppertal, Witten/Herdecke University participated in the clinical observational study. Patients were between 29 and 70 years of age (mean age = 52.91 years, standard deviation [SD] = ± 10.61). All patients received breast-conserving surgery: *N* = 2 patients had postoperative UICC (Union for International Cancer Control, 8th edition 2018) tumor stage 0 (in situ Ca); *N* = 16 patients had stage I, *N* = 5 patients had stage II, and *N* = 2 patients had stage III. Radiotherapy was performed hypofractionated (whole dose 40.05 Gy, single dose 2.67 Gy, 5 times a week) if the target volume covered the breast only (*N* = 18) and normofractionated (whole dose 50.4 Gy, single dose 1.8 Gy, 5 times a week) if the target volumes covered the breast and additionally the lymphatic pathways (*N* = 7). An additional boost to the tumor bed (median 10 Gy; single dose 2 Gy daily) was given in 16 patients. Due to ethical considerations, a control group of patients with breast cancer who had the same testing procedure, but did not receive radiation therapy was not included in the study. Each patient gave written informed consent before study participation. The study is in accordance with the current version of the Declaration of Helsinki of the World Medical Association. It was approved by the Ethics Committee of Witten/Herdecke University. Inclusion criteria were first diagnosis of locoregional breast cancer (ICD-10; C 50.–), age ≥ 18 years, normal or corrected to normal vision, and good knowledge of the German language. Exclusion criteria were age < 18 years, severe medical (e.g., dementia, diabetes, chronic inflammation) and/or psychiatric diseases (e.g., psychosis, personality disorders), and insufficient knowledge of the German language.

Intelligence quotient (IQ) ranged from 80–130, with a mean IQ of 101.28 (SD = 14.23).

Demographic characteristics of the sample are summarized in Table 1.

**Table 1:** Demographic characteristics of the patients (*N* = 25) and frequency distribution of personal status, children, and professions in the study patients

	*N*
*Personal status*	
Married	17
Partnership	4
Divorced/widowed	2
Single	2
*Children*	
Infant age	2
Adolescence	8
Adulthood	10
No children	5
*Profession*	
Salesperson in a bakery shop	1
Banker	1
Nurse for the elderly	1
Housewife	3
Clerk	2
Electronic data processor	1
Secretary	1
Dentist	1
Retiree	1

### Testing instruments

Sociodemographic data were acquired from each patient by a self-constructed questionnaire targeting information on family, cultural background, and chronic diseases (Supplemental information, appendix A1). Anxiety and depression were assessed with several psychological questionnaires covering distinct dimensions of health-related and situational changes of emotional states. For the differential assessment of state and trait anxiety, the State–Trait Anxiety Inventory (STAI, [14]) was applied. The questionnaire allows for separate measures of state anxiety that may vary across time and trait anxiety, which is assumed to represent a rather stable personality trait. Symptoms of depression were assessed with the Beck Depression Inventory-Fast Screen (BDI-FS [15]). The BDI-FS addresses symptoms of depression during the last two weeks. The questionnaire does not allow for a clinical diagnosis of major depression. Rather, it gives information on current and possibly varying symptoms of depression, including suicidal thoughts. Furthermore, the Hamilton Anxiety and Depression Scale (HADS‑D, [16]) was used for additional combined measures of both anxiety and depression. The questionnaire refers to symptoms of anxiety and depression during the last week. It includes fourteen items, with seven addressing depression and seven addressing anxiety such that separate scores can be calculated for each target emotional state. The HADS‑D does not allow for a clinical diagnosis of either major depression or anxiety disorders. To measure rapid changing situational states of affect, the Visual Analogue Emotion Scales (VAMS, [17]) were used. The VAMS addresses eight situational affective states (anxiety, discomfort, sadness, anger, drive, tiredness, happiness, strain) and is particularly suitable to assess situational and daily emotions that may change rapidly across time. Target emotions are presented visually by emoticons and verbally by the respective emotional adjective. Patients indicate on a line drawn from neutral to the target emotion how intense they feel each affective state of interest. The VAMS does not allow for a clinical diagnosis of any emotional disorders. The level of general intelligence was measured with the Mehrfachwahl-Wortschatz test (MWT, [18]). The MWT requires the discrimination between real words and pseudo-words, with ascending difficulty across items. It mainly gives information on the verbal IQ. However, successful accomplishment of the most difficult MWT items also depends on cognitive capacities (e.g., reasoning, monitoring, memory) that are rather independent of verbal abilities.

### Study protocol

We accomplished a pilot clinical observational study with a longitudinal design. At 4 time points (T1–T4), standard and self-constructed questionnaires were applied to assess the course of anxiety and depressive symptoms from diagnosis until the end of radiation therapy. T1 took place immediately before the initial interview (preradiotherapy informative talk and informed consent) with the attending physician. Patients were informed about the study using a cover story to prevent a bias of questionnaire data due to assumptions of social desirability or to the individual demands of each patient’s self-perception. The cover story instructed patients that they are asked to participate in the routine quality management of the clinic, which aims at maximizing the patients’ satisfaction during treatment. After written informed consent was obtained, patients completed the VAMS to screen their situational affective state. T2 took place after the initial interview, which typically lasts 60 min. At T2 patients completed the VAMS, STAI, HADS, BDI, MWT, and the self-constructed questionnaire. T3 took place immediately before the start of radiotherapy, 7–35 days after the initial interview (mean duration of the time interval between the initial interview and the beginning of radiation therapy: 20.4 days; SD = 6.9). Except the measure of intelligence, which should be stable and not affected by radiotherapy all questionnaires applied at T2 were completed again at T3 (mean duration of the time interval between T3 and T4: 29.9 days; SD = 14.7). After the last radiotherapy session (T4), patients completed the same questionnaires which were used at T3. In total, mean duration of study procedures (T1–T4) was 53.6 days (SD = 13.2 days). After T4, the cover story was disclosed, and the patients were informed in detail about the real aims of the study. Afterwards, patients could withdraw their informed consent for participation in the study that they had given on the basis of the cover story. None of the patients made use of this option. Table 2 gives and summarizes the time points of measurement of the study protocol and the questionnaires applied at each time point.

**Table 2:** Overview of time points of measurement (T1–T4) and the questionnaires and testing instruments applied at each single time point of measurement

	Time points of measurement^a^			
	T1	T2	T3	T4
*Timing*	Before the initial interview	After the initial interview	Before the first radiotherapy session	After the last radiotherapy session
*Information, questionnaires, tests*	Informed consent, cover story, VAMS	VAMS, HADS, BDI-FS, STAI, MWT‑B, Self-constructed questionnaire	VAMS, HADS, BDI-FS STAI State, STAI Trait, Self-constructed questionnaire	VAMS, HADS, BDI-FS, STAI, Self-constructed questionnaire

^a^The time frame of T1–T4 is described in detail in the text

### Statistical analysis

A Kolmogorov–Smirnov test revealed that the data were not normally distributed. Therefore, nonparametric statistical tests were applied. Spearman correlations were calculated to detect putative interrelationships between intelligence and social support with the patients’ symptoms of anxiety and depression. For comparisons between subgroups of the sample, the Mann–Whitney test was applied. Correlations and comparisons were calculated for each test and time point of measurement. Moreover, sum scores of anxiety and depression were calculated to gain overall results across the weeks of investigations. The sum score of anxiety included the state and trait subscales of the STAI, the HADS anxiety subscale, and the VAMS anxiety subscale across T1 to T4. The sum score of depression included the BDI-FS and the HADS depression subscale across T1 to T4. Based on 3 items of the self-constructed questionnaire (“I have informed myself about the disease from acquaintances/friends/family”, “I have informed myself extensively about my disease on the internet”, “I am well acquainted with the subject matter of my disease due to my profession”), a sum score of “knowledge of the disease” was also calculated to assess a putative influence of the patients’ information on breast cancer an radiotherapy on their symptoms of anxiety and depression. Due to the use of nonparametric statistical tests and the pilot character of the study, statistical results were not corrected for multiple comparisons.

## Results

### Descriptive results

The patients’ mean IQ was 101.28 (SD = 14.23; range 80–130). The anxiety VAMS scores depict the course of situational anxiety across all 4 time points of measurement. It shows that after the first interview the mean anxiety score decreased from 19.64 (T1) to 16.56 (T2). Immediately before the start of radiation (T3), the anxiety score is highest (27.68). After the end of radiation (T4; recorded directly after the last fraction and the final interview), the anxiety score is the lowest (14.8). Mean scores and SD in each applied anxiety and depression questionnaires for each time point of measurement (*N* = 25) are summarized in Table 3.

**Table 3:** Depression and anxiety in the study patients across T1–T4

	T1		T2		T3		T4	
	Mean	SD	Mean	SD	Mean	SD	Mean	SD
Anxiety HADS	–	–	6.24	3.79	6.36	4.00	5.44	4.23
Anxiety STAI State	–	–	39.16	11.81	43.60	12.73	37.40	10.70
Anxiety STAI Trait	–	–	38.08	10.53	38.84	12.15	40.32	11.38
Anxiety VAMS	19.64	32.95	16.56	20.47	27.68	30.46	14.80	23.36
Depression HADS	–	–	4.20	4.12	4.64	4.20	4.52	4.57
Depression BDI	–	–	1.16	1.41	1.32	1.60	1.68	2.43

Mean HADS, STAI, VAMS, and BDI scores and SD for each time point of measurement (T1–T4). HADS and STAI measures were only taken from T2–T3

*SD* standard deviation

### Relationship of demographic data and measures of anxiety and depression

There was a single significant positive correlation of age and the VAMS anxiety scale at T1 (*r* = 0.474, *p* = 0.026). Correlations of age and sum scores of anxiety were not significant. There was not any significant correlation between age and depression.

Patients who were married or lived in a stable partnership (*N* = 21) had significantly lower scores of anxiety and depression at single time points of measurement than patients who were divorced or lived alone (*N* = 4). At T4, scores in the HADS anxiety subscale were significantly lower in patients who were married or lived in a stable partnership than in patients who were divorced or lived alone (*r* = −2.01, *p* *=* 0.044). At T2, there were nonsignificant tendencies towards the same direction for the HADS anxiety subscale (*r* = −1.79, *p* = 0.073), the VAMS anxiety subscale (*r* = −1.73, *p* = 0.084), and the HADS depression subscale (*r* = −1.95, *p* = 0.052). The existence of one’s own children in any age had no significant influence on anxiety and depression of the patients.

Likewise, there were no significant effects of religiousness.

### Relationship of health-related data and measures of anxiety and depression

**Fig. 1:**
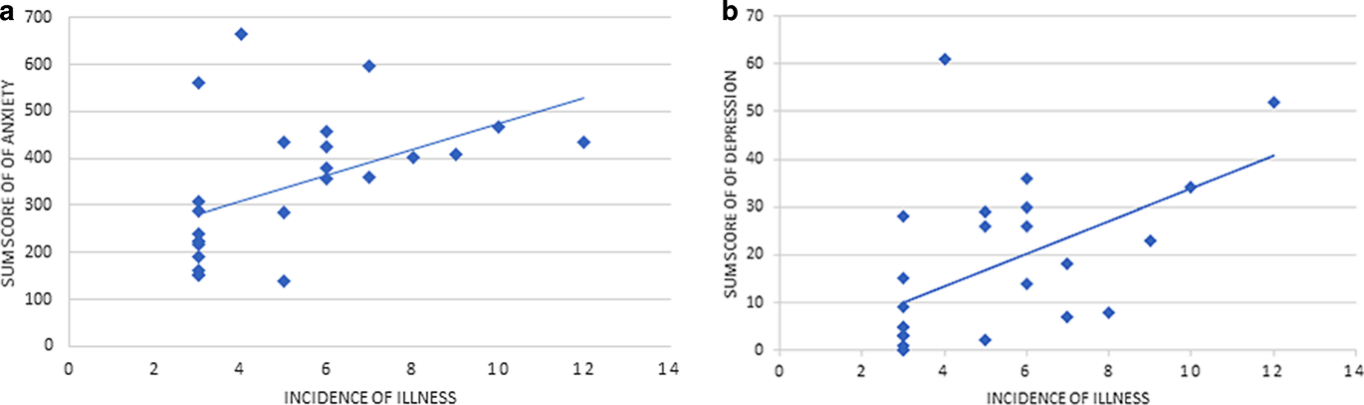
**a** The effect of lifetime incidence of illness on anxiety—relationship between the incidence of illness and anxiety. The x‑axis indicates the number of years of lifetime incidence of any severe illness. The y‑axis indicates the sum scores of anxiety derived from all anxiety questionnaires across all time points of measurement (T1–T4). Higher sum scores indicate higher levels of anxiety. **b** The effect of lifetime incidence of illness on depression—relationship between the incidence of illness and depression. The x‑axis indicates the number of years of lifetime incidence of any severe illness. The y‑axis indicates the sum scores of depression derived from all depression questionnaires across all time points of measurement (T1–T4). Higher sum scores indicate higher levels of depression

There were significant positive correlations between the incidence of illness in the course of the patients’ life (item of the self-constructed questionnaire) and sum scores of anxiety (*r* = 0.607, *p* = 0.001) and depression (*r* = 0.587, *p* *=* 0.002) across all time points of measurement. Correlations are illustrated in Fig. 1. Except T1, similar positive correlations were detected in calculations of single scores in testing instruments for each time point of measurement. The sum score of “knowledge of the disease” did not have any significant effect on anxiety and depression. Likewise, prior experiences with severe diseases in the social environment did not significantly influence symptoms of anxiety and depression in the patients.

### Relationship of intelligence and measures of anxiety and depression

**Fig. 2:**
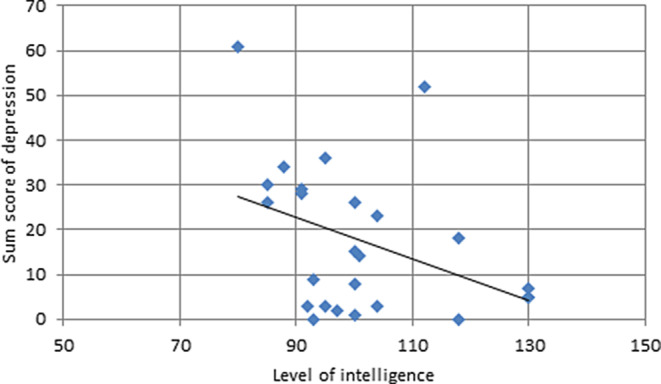
Relationship between intelligence quotient (IQ) and depression. The x‑axis indicates IQ levels. The y‑axis indicates the sum scores of depression derived from depression questionnaires across all time points of measurement (T1–T4). Higher sum scores indicate higher levels of depression

There was no significant relationship between sum scores of anxiety measures across T1–T4 (sum of STAI, HADS, and VAMS scores of anxiety) and IQ (*r* = −0.155, *p* = 0.461). Likewise, correlations between single measures of anxiety at each time point of measurement and IQ were nonsignificant. There was a significant negative correlation (*r* = −0.403, *p* = 0.045) between IQ and sum scores of depression measures (sum of BDI-FS and HADS scores of depression). The correlation is illustrated in Fig. 2. Moreover, there were significant negative correlations of IQ and scores on both the HADS depression subscale (*r* = −0.412, *p* = 0.041) and BDI-FS (*r* = −0.439, *p* = 0.028) at T3. At T4 there was also a significant correlation of IQ and scores on the HADS depression subscale (*r* = −0.418, *p* = 0.038), but not scores on the BDI-FS.

## Discussion

In this pilot clinical observational study we aimed at identifying intelligence-related, health-related, and demographic factors or modulators that may either enhance or reduce the emergence of anxiety and depression in women with breast cancer in the course of radiation therapy. We found that patients had higher levels of anxiety before the initial consultation with the physician than afterwards. Anxiety was strongest immediately before the first radiation treatment. Anxiety and depression were enhanced in women with a lifetime history of chronic diseases. Women with high intelligence and low social support had stronger symptoms of anxiety than women with low intelligence and a stable family background. Patients who were married or lived in a stable partnership had significantly lower scores of anxiety and depression at single time points of measurement than patients who were divorced or lived alone. In contrast, the degree of depression was neither related to intelligence nor to social support. Overall, the level of depression was low across patients. To our knowledge, we for the first time present data on emotional stages of patients with breast cancer during radiation therapy. We propose that results of our study may help to reduce symptoms of anxiety and depression in patients with breast cancer by the implementation of individually optimum clinical procedures for each patient.

### Changes of anxiety across radiation therapy

We saw that patients had higher levels of anxiety before the initial consultation with the physician than afterwards (according to VAMS scores). This underlines the value of the first interview and, in our view, indicates that empathy of the interviewing physician is important. It has been reported for other clinical contexts that empathy is an important factor and has a considerable potential to reduce the patient’s anxiety in the context of a disease [19–21]. Anxiety was strongest immediately before the first radiation treatment. From our point of view a further clinically relevant conclusion can be derived from this finding. In addition to the initial interview an empathetic approach by the physician and other members of the radiation team immediately before the first radiation treatment may have a beneficial influence on the patient’s initial fears. This empathetic approach needs to be adapted individually for each patient. It must be noted here that the variance of our VAMS data at each of the four time points of measurement is relatively large and that our findings need to be corroborated in future studies. Nonetheless, we suppose that our results may give guidance to physicians and the radiation team for the optimization of communication and interaction with the patients.

### Intelligence

Previous data on cognitive factors influencing anxiety and depression in patients with breast cancer are rare. Moreover, available data focusing this issue are inconsistent. Some data indicate a positive correlation between the degree of intelligence and the degree of anxiety and depression. In particular, intelligence has been proposed to exert a significant influence on symptoms of generalized anxiety disorder and symptoms of depression. Coplan et al. found that anxiety and intelligence were positively correlated in patients with a generalized anxiety disorder, together with an inverse relationship between anxiety and intelligence in healthy subjects [22]. Results of a previous study support these data by demonstrating positive correlations between a high degree of worry in patients with generalized anxiety disorder with intelligence [23]. Moreover, Penney et al. found positive correlations between generalized anxiety disorder and verbal intelligence [24]. Results of our study on women with breast cancer contradict these previous findings in that they show a significant negative correlation between intelligence and depression and not any significant relationship between intelligence and anxiety. This divergence of data could be explained at least in part by the investigation of samples with distinct primary diseases. The authors who reported positive correlations between intelligence, anxiety, and depression partly investigated patients with generalized anxiety disorder or also healthy volunteers [23, 24]. In contrast, we assessed oncological patients, whose symptoms of anxiety and depression depended on the oncological diagnosis. It is likely that patients with high intelligence aim at understanding their illness and their chance of survival more deeply (e.g., using the internet and scientific publications) than patients with low intelligence. As a result, detailed negative information on their medical situation may lead to enhanced depressive symptoms. It needs to be further investigated why we did not also find a negative correlation between high intelligence and anxiety in our study patients. Previous data on this issue are rare and inconsistent. Tsaras et al. reported that women with breast cancer who have lower education were more likely to experience anxiety and depression symptoms [5]. A study analyzing the course of anxiety and depression in breast cancer and gynecological cancer patients by Schwarz et al. showed that higher education was associated with higher levels of anxiety at the beginning of treatment [25]. After a few months, the more educated patients showed lower anxiety levels compared to the patients with lower educational levels. The authors argue that this change may be due to a greater ability of higher educated patients to adapt to the disease situation [25]. Note that none of these previous studies related the educational level to the level of intelligence. To our knowledge, no previous data that specifically address issues concerning intelligence, depression, and anxiety in patients with breast cancer have been published. It thus needs to be further investigated whether there may exist a general relationship between high intelligence and the risk of depression and/or anxiety in patients with breast cancer. Future studies have to take into consideration that modulating factors besides intelligence may contribute to the inconsistent pattern of published data concerning this issue [26–28].

### Social factors

We found that women with low social support had stronger symptoms of anxiety than women with a stable family background. Patients who were married or lived in a stable partnership had significantly lower scores of anxiety and depression at single time points of measurement than patients who were divorced or lived alone. This finding corroborates results of previous studies. The phenomenon is well-known and has already been shown by several working groups [29–32]. Puigpinós-Riera et al. performed a cohort study including 1086 women diagnosed with breast cancer and found that low emotional support and social isolation were risk factors for having more anxiety and depression [31]. In our study, social support decreased anxiety, but not depression. It is reasonable to assume that anxiety is influenced to a greater extent and is more sensitive than depression, regarding social support and related feelings of safety. In contrast, depression is likely to evolve slower and rather independent of social support and interaction than does anxiety [33, 34]. This could also be one reason why we found low levels of depression in women shortly after the first diagnosis of breast cancer.

Overall, there is strong evidence that social support is beneficial for the development of successful coping strategies in patients with a diagnosis of breast cancer. To our knowledge, data that differentiate between social support by private dimensions such as family and dimensions of profession such a successful career in distinct kinds of profession are currently unavailable.

### Health-related factors

Moreover, our data show that both anxiety and depression were enhanced in women with a lifetime history of chronic diseases. These findings are supported by the work of Inhestern et al. who found that a better health status was significantly associated with lower depression and anxiety in cancer survivors [35]. Furthermore, Krauss et al. investigated 485 tumor patients using a structured clinical interview and found that lower physical functioning may predispose to anxiety disorders [36]. In our study, a lifetime history of chronic diseases affected not only anxiety, but also depression. However, in this context it is important to note that levels of depression were unexpectedly low in our patient group. Given the severity of the disease we assumed that depression would be more virulent after diagnosis and during radiotherapy. Based on our data, we therefore need to take into consideration that the degree of depression can be modulated by health-related and other factors (e.g., demographic and social data) besides the breast cancer diagnosis itself. Thus, the coping strategies, dimensions of private support, and/or professional situations that may prevent women with breast cancer from becoming depressed need further investigation.

### Limitations of the study

The lack of significance of some correlational relationships in our study is possibly due to the pilot character of the study with a rather small sample size. Because data were not normally distributed, parametric statistical tests could not be performed. Moreover, standard deviations in HADS and STAI data are high (Table 3). Given the small number of patients, we accordingly see some scatter in the anxiety scores in the HADS and STAI in the cohort caused by few outliers (Fig. 1 and 2). Overall, however, the data of this clinical observational study are congruent. Results need to be scrutinized by controlled clinical trials. Due to ethical reasons, the option of including a control group of patients with breast cancer in clinical trials is admittedly impossible or at least limited.

## Conclusion

For the first time, we provide empirical data on cognitive and social modulators of anxiety and depression in a sample of women with breast cancer during radiation therapy. In general, a patient-focused and empathic first interview seems to be of particular importance to diminish anxiety during the course of radiation therapy. Furthermore, to cope with anxiety, the patients need to be approached in a particular individually adapted and empathetic manner immediately before the first irradiation. At this time, understandably, anxiety is at its highest level. Furthermore, our data suggest that medical anamnesis of patients with breast cancer who will be treated with radiotherapy should include details of the individual social situation, measures of intelligence, and further cognitive capacities. We propose that detailed knowledge of the patients’ social background and cognitive characteristics may help physicians and medical-technical assistants to individually adjust and optimize modalities of information on radiotherapy and the interaction with patients during the weeks of treatment. Among other things, women who live alone seem to need more support and encouragement from clinical staff. The recommended offer of psycho-oncological support can be particularly beneficial for these patients. With respect to cognition, our results only demonstrate that the level of intelligence may modulate the degree of anxiety and depression in patients with breast cancer. However, it is likely that other neuropsychological domains such as executive functions and social cognition may also act as modulators of emotional distress during the course of radiotherapy. Future studies that address the interaction between different cognitive functions and facets of social background for the emergence of anxiety and depression in patients with breast cancer undergoing radiotherapy are needed. We expect that our results may help to improve clinical procedures of treatment in these vulnerable patient group to minimize symptoms of anxiety and depression.

## Supplementary Information

Appendix A1
