# Supplementary material for: Anxiety and depression in patients with breast cancer undergoing radiotherapy: the role of intelligence, life history, and social support—preliminary results from a monocentric analysis
Source: Strahlenther Onkol. 2022 Mar 3;198(4):388–96. doi: 10.1007/s00066-022-01904-7 (PMC8940795; doi:10.1007/s00066-022-01904-7)
Supplement: Supplementary file 1 — Appendix A1 [file 66_2022_1904_MOESM1_ESM.docx]

***Self-constructed questionnaires for patients and staff***

**Questionnaire for patients**

Age:

Profession:

**Instructions:** This questionnaire contains statements. Please read each statement carefully and then select the statement that best describes how you have felt in the last two weeks, including today. Tick assertion that applies. Please make sure that you tick only one assertion. Answers cannot be wrong or right.

1. The doctor/staff member took enough time to talk to you.

- I am very satisfied
- I am quite satisfied
- Neither satisfied nor dissatisfied
- Very dissatisfied

1. What impression did the doctor/staff member make on you?

- Competent / well informed
- Open / understandingly / empathic
- Rushed/uncertain
- Arrogant/disintereste

3. Do you think the doctor/staff member feels with you?

- Very much
- Partially
- A little bit
- Not at all

4. Have you shared your fears and concerns about the disease with the doctor/staff member?

- Very
- Partial
- A little
- Not at all

5. Was the doctor/staff member able to reduce your fears about the disease?

- Very
- Partial
- A little
- Not at all

6. I feel that I am in good hands with the doctor/staff regarding the treatment:

- Very good
- Appropriate
- Less good
- Not at all

7. Today I feel:

- Very good
- Appropriate
- Less good
- Bad

8. I am afraid of the course of the disease:

- Often
- Sometimes
- Rather rare
- Never

9. I feel that my actions are slowed down by the illness:

- Often
- Sometimes
- Rather rare
- Never

10. I am afraid:

- Often
- Sometimes
- Rather rare
- Never

11. I ponder and think a lot. There is never peace in my head:

- Often
- Sometimes
- Rather rare
- Never

12. I have panic attacks:

- Often
- Sometimes
- Rather rare
- Never

13. I can relax and switch off well:

- Often
- Sometimes
- Rather rare
- Never

14. I am well acquainted with the subject matter of my disease due to my profession:

- I completely agree
- I mostly agree
- I less agree
- I do not agree at all

15. I am:

- Married
- In a partnership
- Divorced / Widowed
- Single

16. I have children in the:

- Infancy
- Adolescence
- Adulthood
- I have no children

17. I have received support from my family during my illness:

- Often
- Sometimes
- Rather rare
- Never

18. I am rather restrained:

- I agree
- I rather agree
- I less agree
- I do not agree at all

19. I trust others easily, believe in the good in people:

- Applies
- More likely to apply
- Less true
- Does not apply

20. I tend to be lazy:

- Applies
- More likely to apply
- Less true
- Does not apply

21. I am relaxed, I do not let stress upset me:

- Applies
- More likely to apply
- Less true
- Does not apply

22. I have only little interest in art:

- Applies
- More likely to apply
- Less true
- Does not apply

23. I have informed myself extensively about my disease on the internet:

- Applies
- More likely to apply
- Less true
- Does not apply

24. I have informed myself about the disease from acquaintances/friends/family:

- Applies
- More likely to apply
- Less true
- Does not apply

25. I already have experience of serious illness in my social environment:

- Applies
- More likely to apply
- Less true
- Does not apply

26. I have been ill many times in the course of my life:

- Applies
- More likely to apply
- Less true
- Does not apply

27. I believe in a higher power or a god:

- Applies
- More likely to apply
- Less true
- Does not apply

28. I feel that the illness is a punishment from god:

- Applies
- More likely to apply
- Less true

30. The term "cancer" has always frightened me:

- Applies
- More likely to apply
- Less true
- Does not apply

31. I am most afraid of chemotherapy:

- Applies
- More likely to apply
- Less true
- Does not apply, but before

32. I did everything right in terms of therapy selection and treatment:

- Applies
- More likely to apply
- Less true
- Does not apply, what would you have done differently:

**Questionnaire to doctor/medical stuff**

Age:

Profession:

**Instructions:** This questionnaire contains assertions. Please read each assertions carefully and then select a assertions that is most applicable. Tick the assertions that applies. Please make sure that you tick only assertions. There are no right and wrong answers.

1. I took enough time to talk to the patient:

0 I am very satisfied

0 I am quite satisfied

0 Neither satisfied nor dissatisfied

1. Very dissatisfied

2. What impression did the patient make on you?

0 Well informed

0 Open/hopeful

0 Nervous/uncertain

0 Hopeless/Dejected

3. Do you have fears and concerns about the patient's disease course?

- Very
- Partial
- A little
- Not at all

4. I can meet the patient's needs in terms of treatment:

- Very good
- Appropriate
- Less good
- Not at all

5. I feel today:

- Very good
- Appropriate
- Less good
- Bad

6. I am afraid:

- Often
- Sometimes
- Rather rare
- Never

7. I can relax and switch off well:

- Often
- Sometimes
- Rather rare
- Never

8. I have children in the:

- Primary school age
- Adolescence
- Adulthood
- I have no children

9. I am rather restrained:

- Applies
- More likely to apply
- Less true
- Does not apply

10. I trust others easily, believe in the good in people:

- Applies
- More likely to apply
- Less true
- Does not apply

11. I am relaxed, I do not let stress upset me:

- Applies
- More likely to apply
- Less true
- Does not apply

12. I have read the patient's medical reports thoroughly:

- Applies
- More likely to apply
- Less true
- Does not apply

13. I have checked with colleagues about the patient:

- Applies
- More likely to apply
- Less true
- Does not apply
